# Medical Selections from Herndon’s Report on the Valley of the Amazon

**Published:** 1854-06

**Authors:** 


### *Medical Selections from Herndon's Report on the Valley of the Amazon.*

The boats of the party had sprung leaks and it became necessary, on the score of safety, to re-calk them. But where could pitch be found in that region of savage wildness. The following extract explains it:

"The pitch of the country is said to be the deposit of an ant in the trees. I never saw it in its original state. It is gathered by the Indians; heated till soft; made into the shape of wide, thin bricks; and is worth sixty-two and a half cents the arroba. It is very indifferent. A better kind is made by mixing black wax with gum copal."

He speaks of an epidemic catarrh among the inhabitants, its symptoms and of his prescriptions as follows:

"A number of persons were affected with catarrh and headache. The padre told me that half of the population were ill of it, and that this always happens at the commencement of the rains. The disease is called *romadiza,* and is like our influenza. Ijurra and I were both indisposed with rheumatic pains in the back of the neck and shoulders. I gave the padre's servant, who was suffering very much from romadiza, fifteen grains of Dover's powder, (Heaven knows if it were proper or not,) and also to the padre's sister, who had been suffering for some days with painful diarrhœa, forty drops of laudanum. The old lady was cured at once, and said she had never met with so great a remedio. I left her a phial of it, with directions for its use; telling her (at which she looked aghast) that it was a deadly poison. It is curious to see how entirely ignorant the best informed people out here are concerning the properties of medicines. Most of them do not know the names, much less the effects, of even such common drugs as calomel and opium. I suspect this is the case among most Spanish people, and think that Spanish physicians have always made a great mystery of their science."

The following description might find a fitting parallel in the mighty gatherings of the believers in "spirit rappings" of this day of enlightenment, with the difference only of the modifications in the sounds and the motives of those who hold intercourse with the spirits. The savage resorts to it from humane motives, while the "spirituals" of the present day are moved by curiosity alone.

"After dark he proposed that we should go out and see some of the incantations of the Indians for the cure of the sick. We heard music at a distance, and approached a large house whence it proceeded, in which the padre said there was almost always some one sick. We listened at the door, which was closed. There seemed to be a number of persons singing inside. I was almost enchanted myself. I never heard such tones, and think that even instrumental music could not be made to equal them. I have frequently been astonished at the power of the Indians to mock animals ; but I had heard nothing like this before. The tones were so low, so faint, so guttural, and at the same time so sweet and clear, that I could scarcely believe they came from human throats ; and they seemed fitting sounds in which to address spirits of another world."

Of the character and variety of the different dyes of the country, he says :

"I noticed growing about the houses of the village a couple of shrubs, six or eight feet high, called respectively, *yanapanga* and *pucapanga.* From the leaves of the first is made a black dye, and from those of the second, a very rich scarlet. I surmised that a dye, like the indigo of commerce, though of course of different color, might be made of these leaves ; and when I arrived in Brazil, I found that the Indians there were in the habit of making a scarlet powder of the pucapanga, called carajura, quite equal, in brilliancy of color, to the dye of the cochineal. I believe that efforts have been made to introduce this dye into commerce, and I do not know why they have failed. I brought home a specimen."

He speaks of a species of blistering fly that answered well for vesication.

"I was told that Castelnau found here a fly that answered perfectly all the purposes of cantharides, blistering the skin even more rapidly. I heard that he also found the same fly at Egas, lower down. Senhor Lima instituted a search for some for me, but there were none to be had at this season."

On Christmas he arrived at Egas, which by reference to the map, we find to be at, or a little below the mouth of the river Teffee in Brazil. Here the Brazilians own negroes as slaves, whose services are valuable to them. It would appear that from some cause they do not succeed with them, being peculiarly obnoxious to a disease of which they die.

"Major Batalha tells me he has lost six tapuios, by a sort of bloody flux, within the last two months. I asked him if the disease was confined to his his household ; but he told me that it was general, and supposed that it was caused by drinking the water of the lake, which was thought to be, in some small degree, impregnated with the poisonous milk of the assacu, (the Peruvian catao,) many of which trees grow on its borders. I have no idea that this is the cause, but suppose the disease originates from exposure, bad food, and an imprudent use of fruit, though I see no fruit except a few oranges and limes. It is even difficult to purchase a bunch of bananas. There are no other diseases in Egas except tertiana, caught in gathering sarsaparilla on the tributaries."

At Barre, lower down, the Indians are far removed from civilization, and kept in a most degraded condition as slaves. The following description shows this and also that the females here suffer little in parturition.

"They are thorough savages, and kill a number of their children from indisposition to take care of them. My good hostess told me that her father, returning from a walk to his house in the country, heard a noise in the woods; and, going towards the spot, found a young Indian woman, a tapuio of his, digging a hole in the ground for the purpose of burying her infant just born. He interfered to prevent it, when she flew at him like a tiger. The old gentleman, however, cudgelled her into submission and obedience, and compelled her to take the child home, where he put it under the care of another woman."

The following indicates the botanical resources of this country, and confirms what we have said before, that this expedition should have embraced a full scientific corps.

"There are twelve kinds of trees that exude milk from their bark; the milk of some of these—such as the arvoeiro and assacu—is poisonous. One is the seringa, or India-rubber tree; and one the *murure,* the milk of which is reported to possess extraordinary virtue in the cure of mercurialized patients, or those afflicted with syphilitic sores. Mr. Norris told me that a young American, dreadfully afflicted with the effects of mercury, and despairing of cure, had come to Para to linger out what was left of life in the enjoyment of a tropical climate. A few doses of the murure sent him home a well man, though it is proper to say that he died suddenly a few years afterwards. Captain Littlefield, the master of the barque "Peerless," told me that he had a seaman on board his vessel covered with sores from head to foot, who was radically cured with a few teaspoonfuls of murure. Its operation is said to be very powerful, making the patient cold and rigid, and depriving him of sense for a short time. Mr. Norris has made several attempts to get it home, but without success. A bottle which I brought had generated so fœtid a gas that I was glad to toss it from my hand when I opened it at the Observatory.

It is idle to give a list of the medicinal plants, for their name is legion. The Indians use nearly everything as a "remedio." One, however, is peculiar—it is called *manaca.* Von Martius, a learned German, who spent several years in this country, thus describes it: "Omnis planta, magna radix potissimum, systema lymphaticum summa efficacia excitat, particulas morbificas liquescit, sudore et urina eliminat. Magni uses in syphilitide, ideo *mercurio vegetal* a quibusdam dicitur. Cortex interior et omnes partes herbaceæ amaritudine nauseosa, fauces vellicante, pollent. Dosi parva resolvit, majore exturbat alvum et urinam ciet, abortum movet, venenum a morsu serpentum excutit; nimia dosi tanquam venenum acre agit. De modo, quo hauriri solet, conferas Martium, in Buchner Reporter Pharm. XXXI, 379. Apud nonullas Indorum gentes in regione Amazonica habitantes ejus extractum in venenum sagittarum ingreditur."

Its virtue in rheumatic affections was much extolled; and, as I was suffering from pains in the teeth and shoulders, I determined to try its efficacy; but, understanding that its effects were powerful, and made a man feel as if a bucket of cold water were suddenly poured down his back, I begged my kind hostess, Donna Leocadia, to make the decoction weak. Finding no effects from the first teacupful, I took another; but either I was a peculiar patient, or we had not got hold of the proper root. I felt nothing but a very sensible coldness of the teeth and tip of the tongue. Next morning I took a stronger decoction, but with no other effect. I think it operated upon the liver, causing an increased secretion of bile. I brought home the leaves and root.

The root of the *murapuama,* a bush destitute of leaves, is used as an analeptic remedy, giving force and tone to the nerves.

A little plant called *douradinha,* with a yellow flower something like our dandelion, that grows in the streets at Barra, is a powerful emetic.

A clear and good-burning oil is made from the Brazil nut; also from the nut of the andiroba, which seems a sort of bastard Brazil nut, bearing the same relation to it that our horse-chesnut does to the edible chesnut. Both these oils, as also the oil made from turtle-eggs, are used to adulterate the copaiba. The trader has to be on the alert that he is not deceived by these adulterations. Another very pretty oil or resin is called *tamacuare*; its virtues are much celebrated for the cure of cutaneous affections."

We will conclude our extracts in the next, in which we will call the attention of the reader to an important fact or two in relation to the opinion of the unity of the races, and the extraordinary power of certain remedies used by the natives.

